# Drivers of COVID-19 policy stringency in 175 countries and territories: COVID-19 cases and deaths, gross domestic products per capita, and health expenditures

**DOI:** 10.7189/jogh.12.05049

**Published:** 2022-12-17

**Authors:** Mohamed F Jalloh, Zangin Zeebari, Sophia A Nur, Dimitri Prybylski, Aasli A Nur, Avi J Hakim, Maike Winters, Laura C Steinhardt, Wangeci Gatei, Saad B Omer, Noel T Brewer, Helena Nordenstedt

**Affiliations:** 1 Center for Global Health, U.S. Centers for Disease Control and Prevention, Atlanta, Georgia, USA; 2 Department of Global Public Health, Karolinska Institute, Stockholm, Sweden; 3 Jönköping International Business School, Jönköping University, Jönköping, Sweden; 4 Department of Sociology, University of Washington, Seattle, Washington, USA; 5 Institute for Global Health, Yale University, New Haven, Connecticut, USA; 6 Department of Health Behavior, Gillings School of Global Public Health, University of North Carolina, Chapel Hill, North Carolina, USA; 7 Lineberger Comprehensive Cancer Center, University of North Carolina, Chapel Hill, North Carolina, USA; 8 Department of Internal Medicine and Infectious Diseases, Danderyd University Hospital, Stockholm, Sweden

## Abstract

**Background:**

New data on COVID-19 may influence the stringency of containment policies, but these potential effect are not understood. We aimed to understand the associations of new COVID-19 cases and deaths with policy stringency globally and regionally.

**Methods:**

We modelled the marginal effects of new COVID-19 cases and deaths on policy stringency (scored 0-100) in 175 countries and territories, adjusting for gross domestic product (GDP) per capita and health expenditure (% of GDP), and public expenditure on health. The time periods examined were March to August 2020, September 2020 to February 2021, and March to August 2021.

**Results:**

Policy response to new cases and deaths was faster and more stringent early in the COVID-19 pandemic (March to August 2020) compared to subsequent periods. New deaths were more strongly associated with stringent policies than new cases. In an average week, one new death per 100 000 people was associated with a stringency increase of 2.1 units in the March to August 2020 period, 1.3 units in the September 2020 to February 2021 period, and 0.7 units in the March to August 2021 period. New deaths in Africa and the Western Pacific were associated with more stringency than in other regions. Higher health expenditure as a percentage of GDP was associated with less stringent policies. Similarly, higher public expenditure on health by governments was mostly associated with less stringency across all three periods. GDP per capita did not have consistent patterns of associations with stringency.

**Conclusions:**

The stringency of COVID-19 policies was more strongly associated with new deaths than new cases. Our findings demonstrate the need for enhanced mortality surveillance to ensure policy alignment during health emergencies. Countries that invest less in health or have a lower public expenditure on health may be inclined to enact more stringent policies. This new empirical understanding of COVID-19 policy drivers can help public health officials anticipate and shape policy responses in future health emergencies.

Policy responses during the COVID-19 pandemic have a dynamic relationship with epidemiological outcomes [1,2]. The effects of public health policies on slowing the spread of severe acute respiratory syndrome coronavirus 2 (SARS-CoV-2) are demonstrated in multiple studies, with new evidence emerging [3-5]. Public health policies to contain the spread of SARS-CoV-2 have included restricting population movements and gatherings, closing schools and businesses, and requiring masks indoors – these policies are also known as non-pharmaceutical interventions (NPIs). In the COVID-19 pandemic, NPIs have been implemented using a combination of top-down, intermediary, and bottom-up approaches [6]. Numerous platforms track the stringency of policies enacted globally since the early stages of the pandemic [7]. Most COVID-19 containment policies implemented in the early stages of the pandemic throughout 2020 primarily used top-down approaches [6]. However, the drivers of COVID-19 containment policies remain poorly understood.

Mathematical modelling suggests that policies for NPI are critical to contain the COVID-19 pandemic alongside improved access to and uptake of COVID-19 vaccines [8]. The emergence of variants of concern with immune evasion capability (such as Omicron) reinforces the need to continually (re)calibrate public health policies to control the pandemic [9]. These modelling findings are layered on the backdrop of persistent inequities in access to and uptake of COVID-19 vaccines in low-income countries compared to middle- and high-income countries [10].

The World Health Organization (WHO) member states report new COVID-19 cases and deaths to the WHO [11]. The reported epidemiological trends create dynamic perceptions about the intensity of the COVID-19 situation in a country [12]. They may also trigger policies that are not aligned with the COVID-19 epidemiology. Moreover, seroprevalence assessments of SARS-CoV-2 have uncovered gross underestimation of the population-level burden of infection when compared to officially reported new cases [13,14]. Variations in testing capacity, testing strategies, and people’s willingness to be tested contribute to the underestimation of SARS-CoV-2 infections [15]. COVID-19-related mortality may also be underreported due to weak mortality surveillance systems, inadequate systems for civil registration of deaths, and different classifications of the causes of death [16].

Socioeconomic conditions and political considerations may also affect the aggressiveness and expediency of a country's response to reported new cases and deaths [17,18]. The WHO recommends that the stringency of containment policies should be “based primarily on a situational assessment of the intensity of transmission and the capacity of the health system to respond but must also be considered in light of the effects these measures may have on the general welfare of society and individuals” [19]. An empirical understanding of the reported epidemiological data’s influence on policy stringency can help predict policy responses in the current pandemic and other health emergencies. To this end, we aimed to model the associations of reported COVID-19 new cases and deaths with policy stringency.

## METHODS

We conducted a policy analysis to understand how changes in the reported new COVID-19 cases and deaths were associated with the stringency of governments’ policies in 175 countries and territories representing all regions globally. We used fractional regression to model the marginal effects of new cases and deaths on COVID-19 policy stringency, adjusting for the gross domestic product (GDP) per capita, health expenditure as a percentage of GDP, and the percentage of public expenditure on health (compared to private and/or external expenditure). We adjusted for GDP per capita because prior studies have shown a dynamic relationship between GDP and policy stringency [20]. Policy stringency may vary dependant on a country’s GDP and government. Protracted “lockdown” policies, for instance, may not be tenable in low GDP countries. We adjusted for health expenditure as a percentage of GDP because countries with greater health investments may have greater investments in their COVID-19 response [21]. The GDP per capita, health expenditure, and level of public expenditure on health do not change remarkably on an annual basis for the same country. Lastly, we adjusted for public expenditure on health because countries with greater government spending on health may behave differently than countries with greater private and/or external spending on health. We used the latest reported GDP, health expenditure as a share of GDP, and public expenditure on health statistics from the United Nations and WHO [22,23].

### Main outcome

Our main outcome was the stringency of COVID-19 policies as measured daily by the Oxford Tracker [24]; its data collection and scoring methods have been described elsewhere.[25] In summary, the stringency index ranges from 0 to 100 as per the original index developed by Oxford University, with higher scores representing more stringent policies. The stringency score for each country or territory is based on nine component indicators measured on an ordinal scale to assess 1) closing of schools and universities, 2) closing of workplaces, 3) cancelling public events, 4) limits on gatherings, 5) closing of public transport, 6) mandates for shelter-in-place/home confinement, 7) restrictions on internal movement between cities/regions, 8) restrictions on international travel, and 9) presence of COVID-19 public information campaigns.

### Statistical analysis

We analysed the data in Stata version 17 SE (StataCorp LLC, College Station, TX) by examining three six-month periods separately for March to August 2020, September 2020 to February 2021, and March to August 2021. These three periods were selected a priori, and the start period (March 2020) was chosen because most countries reported their first COVID-19 case during that time.

**Figure 1:**
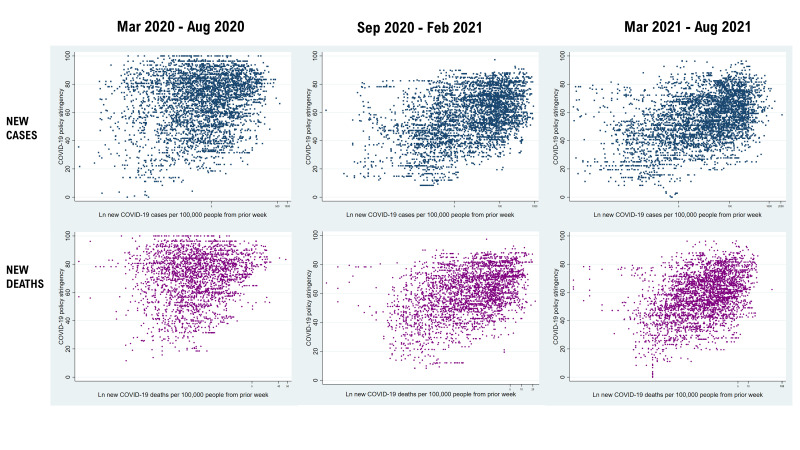
New COVID-19 cases/deaths and policy stringency across 175 countries and territories, March 2020 to August 2021.

First, we described each of the nine ordinally measured stringency indicators in the Oxford Stringency Index for each period and the combined periods (**Table 1**). We then merged the daily COVID-19 epidemiological data countries reported to the WHO [26] with their policy stringency data [24]. New cases and deaths were calculated to reflect cases and deaths per 100 000 people. The daily merged data for cases, deaths, and stringency were aggregated weekly. We used scatter plots to visually describe the changes in the stringency score as the number of reported new cases and deaths per 100 000 people increased globally (**Figure 1**) and regionally (**Online Supplementary Document**) on a logarithmic scale.

**Table 1:** Stringency of specific COVID-19 policies – 175 countries and territories, March 2020 to August 2021*

**Stringency of COVID-19 policy**	March 2020 to August 2020; 32 200 daily reports (median = 68.52)	September 2020 to February 2021; 31 675 daily reports (median = 56.48)	March 2021 to August 2021; 34 040 daily reports (median = 54.63)	Overall – 115 325 daily reports (median = 55.56)
**(C1) Closings of schools and universities**				
0 – no measures	11.19	10.45	20.07	22.07
1 – recommend closing or all schools open with alterations	9.08	34.71	34.42	23.52
2 – require closing for some levels or categories	18.58	26.11	24.43	20.80
3 – require closing all levels	61.15	28.72	20.82	33.52
**(C2) Closings of workplaces**				
0 – no measures	21.03	16.29	14.83	25.17
1 – recommend closing or remote work or significant alterations	13.98	24.34	27.90	20.04
2 – require closing (or remote work) for some sectors or workers	46.02	48.03	45.21	41.99
3 – require closing (or remote work) for all but essential workplaces	18.97	11.30	11.82	12.72
**(C3) Cancelling of public events**				
0 – no measures	13.86	11.50	10.71	20.45
1 – recommend cancelling	13.87	27.85	32.88	22.41
2 – require cancelling	72.26	60.65	56.38	57.12
**(C4) Limits on gatherings**				
0 – no restrictions	18.71	12.62	11.57	22.39
1 – restrictions on very large gatherings of above 1000 people	2.68	2.66	2.87	2.46
2 – restrictions on gatherings between 101 and 1000 people	10.05	11.06	6.66	8.16
3 – restrictions on gatherings between 11 and 100 people	28.28	30.61	31.72	27.26
4 – restrictions on gatherings of 10 people or less	40.28	43.01	47.10	39.68
**(C5) Public transportation restrictions**				
0 – no measures	47.09	57.71	51.52	56.72
1 – recommend closing or significantly reduced	30.84	33.59	39.92	31.42
2 – require closing or prohibit most citizens from using it	22.06	8.62	8.46	11.81
**(C6) Stay-at-home requirements**				
0 – no measures	31.31	31.58	30.15	37.47
1 – recommend not leaving house	26.60	27.34	25.89	24.11
2 – require not leaving house with exceptions for daily exercise, grocery shopping, and 'essential' trips	35.34	37.70	40.20	34.22
3 – require not leaving house with minimal exceptions	6.75	3.34	3.48	4.11
**(C7) Restrictions on internal movement between cities/regions**				
0 – no measures	34.01	47.17	50.63	49.40
1 – recommend not to travel between regions/cities	17.10	17.61	17.02	15.48
2 – internal movement restrictions in place	48.89	35.21	32.05	35.02
**(C8) Restrictions on international travel for foreign travellers**				
0 – no restrictions	5.71	0.30	0.44	9.35
1 – screening arrivals	4.97	22.34	25.00	16.86
2 – quarantine arrivals from some or all regions	11.26	27.97	27.06	20.06
3 – ban arrivals from some regions	28.52	33.05	32.01	29.23
4 – ban on all regions or total border closure	49.53	16.32	15.35	24.46
**(H1) Presence of public information campaigns**				
0 – no COVID-19 public information campaign	3.03	0.30	0.93	8.23
1 – public officials urging caution about COVID-19	5.32	5.89	6.31	6.37
2 – Coordinated public information campaigns	91.65	93.79	92.35	85.26

*Values reported in percentages.

Second, we added each country’s time-invariant data on GDP per capita [27], health expenditure as a share of GDP [22], and public expenditure on health to the data set [23]. Finally, we used fixed-effects fractional regression with a quasi-likelihood method to model the marginal effects [28] of these covariates on policy stringency. We estimated the marginal effects associated with one new case per 100 000 people, one new death per 100 000 people, 1% larger GDP per capita, 1% additional share of GDP spent on health, and 1% additional public expenditure on health. We reported marginal effects instead of regression coefficients to enable more meaningful interpretations of the predicted change in the stringency score. Our models used temporal lags to understand how rapidly governments enacted policies following new cases and deaths. We fit separate models for the marginal effects based on one- and four-week lags between reporting a new case or death per 100 000 people and the associated average change in the stringency. To examine regional differences, we conducted separate analyses for each of the six WHO regions: Africa, Americas, Eastern Mediterranean, Europe, South-East Asia, and Western Pacific. A *P*-value of less than 0.05 was considered statistically significant in all models.

We fit our fixed-effects fractional regression models using a quasi-likelihood method with a logit link to examine associations between weekly new cases/deaths and policy stringency, adjusting for GDP per capita and health expenditure in 175 countries and territories. Our fractional regression models accounted for a country’s specific and time-varying policy stringency on a nonlinear-trend basis by including interaction terms for 1) country × time and 2) country × time^2^. The time-varying effect was measured weekly over six-month periods. The time was mean-centred to avoid multicollinearity between time and time^2^. The mean centring was done by subtracting the middle week number of the studied period from each week number. The country-specific intercept in the fixed-effects model was replaced by time-invariant GDP per capita, health expenditure, and public spending on health at the country level to avoid the dummy trap problem (ie, perfect multicollinearity).

The Oxford Stringency Index [29] scaled in the interval (0,1), for country *i* at time *t* was modelled as:



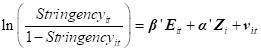



where *E_it_* is the vector of weekly COVID-19 cases and deaths, *Z_i_* the vector of observed and time-invariant country-specific GDP per capita, health expenditure, and public expenditure on health, and *ꞵ* and α are unknown regression parameters to be estimated. Also, *v_it_* is the unobservable time-varying country’s specific performance at time *t*, modelled as:

*v_it_* = *γ_0_* _+_ *γ_1i_* × *t* + *γ_2i_* × *t^2^*

with unknown parameters *γ_0_* , *γ_1i_*, and *γ_2i_*, to be estimated for country *i*. Alternatively, the above country-specific performance at time *t* is presented as in the fixed-effects notation. It can be equivalently represented as:



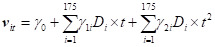



Where *D_i_* is the *i*-th country dummy variable.

For sensitivity analyses, the time-invariant predictors are used in a random-effects beta regression model, using glmmTMB library in R statistical software package version 1.1.2.3 (R Foundation for Statistical Computing, Vienna, Austria). The beta regression model was fitted as:



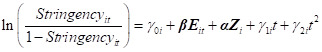



…with *γ_ji_* = *γ_ji_* + *u_ji_*, *j* = 0,1,2 and *u_ji_* a random variable.

The results of the beta regression and the fractional regression were consistent whenever the beta regression is applicable. The beta regression was not appropriate for extreme situations with 0 or 100 stringency scores (0,1 on a fractional scale). Such extreme situations were rare in the data set, especially after the March to August 2020 period. We only report here the results from the fractional regression.

## RESULTS

Policy stringency waned over time. For instance, all levels of schools and universities were required to close on 61.2% of the country-days during March to August 2020 compared to 20.8% during March to August 2021. However, some policies remained relatively unchanged. Restrictions on gatherings between 11 and 100 people were in place for 28.3% of the country-days in the March to August 2020 period compared to 31.7% in March to August 2021. The global median stringency score was higher in the March to August 2020 period (68.5) compared to September 2020 to February 2021 (56.4) and March to August 2021 (54.6) periods (**Table 1**). The unadjusted stringency score increased as new cases and deaths per 100 000 people increased (**Figure 1** and Figures S1 to S6 in **Online Supplementary Document**).

### Global findings

Within one week on average, one new case per 100 000 people was associated with a stringency increase of 0.11 units (95% confidence interval (CI) = 0.08 to 0.13) in the March to August 2020, 0.02 units (95% CI = 0.01 to 0.02) in the September 2020 to February 2021 period, and 0.02 units (95% CI = 0.01 to 0.03) in the March to August 2021 period. In the early pandemic period (March to August 2020), an increase in new cases was associated with more rapidly enacted stringent policies (**Table 2**).

**Table 2:** Marginal effects of correlates on COVID-19 policy stringency in 175 countries and territories, March 2020 to August 2021

Correlates	March 2020 to August 2020*				September 2020 to February 2021*			March 2021 to August 2021*		
	ME	95% CI			ME	95% CI		ME	95% CI	
**One-week lag between reporting of new cases/deaths and policy stringency**										
New cases†	0.10§	0.07	0.13		0.01§	0.01	0.02	0.02§	0.01	0.03
New deaths†	2.01§	1.26	2.76		1.28§	0.94	1.62	0.76§	0.39	1.14
GDP per capita‡	0.00	-0.01	0.01		0.05§	0.04	0.05	0.03§	0.03	0.04
Health expenditure‡	-1.33§	-1.64	-1.02		-0.87§	-1.13	-0.62	-0.84§	-1.07	-0.61
Public expenditure on health‡	-0.24§	-0.30	-0.17		-0.17§	-0.21	-0.12	-0.13§	-0.17	-0.08
**Four-week lag between reporting of new cases/deaths and policy stringency**										
New cases†	0.04§	0.03		0.06	0.01§	0.01	0.02	0.02§	0.02	0.03
New deaths†	0.85§	0.45		1.26	1.15§	0.78	1.52	0.27§	0.02	0.52
GDP per capita‡	0.01§	0.00		0.02	0.05§	0.04	0.06	0.03§	0.03	0.04
Health expenditure‡	-1.09§	-1.35		-0.83	-0.76§	-1.03	-0.50	-0.83§	-1.07	-0.59
Public expenditure on health‡	-0.21§	-0.27		-0.16	-0.16§	-0.21	-0.11	-0.13§	-0.18	-0.08

ME – marginal effects from three separate fractional regression models for each 6-mo period, GDP – gross domestic product, CI – confidence interval

*Epi-week 10 to 35 in 2020 used for March 2020 to August 2020 period; epi-week 36 in 2020 to epi-week 8 in 2021 used for September 2020 to February 2021 period; epi-week 9 in 2021 to epi-week 34 in 2021 used for March 2021 to August 2021 period.

†One new case per 100 000 population; one new death per 100 000 population.

‡1% increase in GDP per capita; 1% additional share of GDP on health expenditure; 1% additional share of public expenditure on health.

§*P* < 0.05.

Within one week on average, one new death per 100 000 people was associated with a stringency increase of 2.1 units (95% CI = 1.3 to 2.9) in the March to August 2020 period, 1.3 units (95% CI = 0.9 to 1.6) in the September 2020 to February 2021 period, and 0.7 units (95% CI = 0.4 to 1) in the March to August 2021 period. As for new cases in the early pandemic period, an increase in new deaths was associated with more rapidly enacted stringent policies (**Table 2**).

Although 1% larger GDP per capita was associated with less stringency in the March to August 2020 period, it was associated with more stringency in the subsequent periods. A 1% additional share of GDP spent on health was associated with less stringency across all three periods. Moreover, a 1% additional public expenditure on health as fraction of the total health expenditure was associated with less stringency across all three periods (**Table 2**).

### Regional findings

We show the regional-specific marginal effects on policy stringency for a one-week lag (**Table 3**) vs a four-week lag (**Table 4**) after reporting new cases and deaths. An average increase of one new case per 100 000 people was associated with more stringent policies within one week in the Americas and Europe across all three periods. There was a slow policy response to new cases in Africa early in the pandemic. However, the association between new deaths and stringency was strongest in Africa and the Western Pacific early in the pandemic. Eastern Mediterranean was the only region where the new cases were ever associated with less stringency (March to August 2021). New deaths were only associated with less stringency in Eastern Mediterranean (March to August 2020) within a one-week lag and in Europe (March to August 2020) within a four-week lag.

**Table 3:** Regional marginal effects of correlates on COVID-19 policy stringency in 175 countries and territories, based on a one-week lag after the reporting of cases and deaths, March 2020 to August 2021

Correlates	Africa			Americas			East Mediterranean			Europe			South-East Asia			Western Pacific		
	ME	95% CI		ME	95%CI		ME	95% CI		ME	95% CI		ME	95% CI		ME	95% CI	
March 2020 to August 2020*																		
New COVID-19 cases†	-0.01	-0.15	0.13	0.10§	0.01	0.19	0.01	-0.01	0.03	0.12§	0.06	0.18	0.99	-0.21	2.19	0.02	-0.06	0.10
New COVID-19 deaths†	25.18§	15.19	35.17	-0.03	-0.79	0.73	-8.57§	-14.00	-3.14	1.98§	0.89	3.07	22.55	-36.42	81.52	96.60§	34.02	159.17
GDP per capita‡	0.09§	0.06	0.12	0.00	-0.04	0.03	-0.01	-0.04	0.03	-0.03§	-0.05	-0.02	0.00	-0.11	0.10	0.03§	0.02	0.05
Health expenditure‡	-0.47	-1.07	0.14	-1.60§	-2.43	-0.77	-1.60§	-2.71	-0.50	-1.10§	-1.64	-0.55	5.54	-0.28	11.35	-3.02§	-3.70	-2.35
Public expenditure on health‡	-0.48§	-0.62	-0.34	-0.11	-0.23	0.00	0.02	-0.19	0.23	-0.07	-0.20	0.06	-0.24	-0.55	0.07	-0.41§	-0.54	-0.28
September 2020 to February 2021*																		
New COVID-19 cases†	0.11§	0.06	0.17	0.03§	0.01	0.05	0.08§	0.05	0.11	0.01§	0.00	0.01	0.09	-0.13	0.31	0.66§	0.44	0.87
New COVID-19 deaths†	-0.17	-2.09	1.76	0.98	-0.05	2.02	5.34§	3.24	7.44	0.89§	0.59	1.19	3.74	-11.39	18.87	13.36	-2.20	28.92
GDP per capita‡	0.02	-0.01	0.04	0.06§	0.04	0.08	0.01	-0.01	0.03	0.04§	0.03	0.05	0.02	-0.05	0.10	0.04§	0.02	0.05
Health expenditure‡	-1.34§	-1.78	-0.89	-1.75§	-2.28	-1.23	-1.52§	-2.58	-0.47	-0.50§	-0.93	-0.06	-5.87§	-8.19	-3.55	-0.67§	-1.15	-0.19
Public expenditure on health‡	-0.08	-0.20	0.04	-0.07	-0.14	0.01	0.11	-0.06	0.28	-0.25§	-0.33	-0.16	-0.35§	-0.58	-0.11	-0.39§	-0.45	-0.32
March 2021 to August 2021*																		
New COVID-19 cases†	0	-0.01	0	0.03§	0.02	0.04	-0.02§	-0.03	-0.01	0.03§	0.02	0.03	0.14§	0.07	0.20	0.05§	0.01	0.09
New COVID-19 deaths†	1.06§	0.38	1.74	0.23	-0.07	0.53	3.30§	2.19	4.41	-0.15	-0.42	0.11	0.87	-1.66	3.39	-0.18	-4.58	4.22
GDP per capita‡	0.08§	0.06	0.10	0.07§	0.05	0.09	0.01	-0.01	0.03	0.00	-0.01	0.01	-0.07§	-0.12	-0.02	0.03§	0.02	0.05
Health expenditure‡	0.78§	0.32	1.24	-1.33§	-1.97	-0.70	-0.92§	-1.64	-0.20	0.61§	0.25	0.97	-5.40§	-7.61	-3.20	-0.61	-1.25	0.02
Public expenditure on health‡	0.03	-0.06	0.13	-0.02	-0.13	0.09	0.08	-0.04	0.20	-0.09§	-0.15	-0.03	-0.09	-0.21	0.04	-0.73§	-0.81	-0.65

ME – marginal effects from three separate fractional regression models for each 6-mo period, GDP – gross domestic product, CI – confidence interval

*Epi-week 10 to 35 in 2020 used for March 2020 to August 2020 period; epi-week 36 in 2020 to epi-week 8 in 2021 used for September 2020 to February 2021 period; epi-week 9 in 2021 to epi-week 34 in 2021 used for March 2021 to August 2021 period.

†One new case per 100 000 population; one new death per 100 000 population.

‡1% increase in GDP per capita; 1% additional share of GDP on health expenditure; 1% additional share of public expenditure on health.

§*P* < 0.05.

**Table 4:** Regional marginal effects of correlates on COVID-19 policy stringency in 175 countries and territories, based on a four-week lag after the reporting of cases and deaths, March 2020 to August 2021

**Correlates**	Africa			Americas			East Mediterranean			Europe			South-East Asia				Western Pacific		
	ME	95% CI		ME	95% CI		ME	95% CI		ME	95% CI		ME	95% CI		ME		95% CI	
March 2020 to August 2020*																			
New COVID-19 cases†	0.04§	0.03	0.06	0.17§	0.01	0.34	0.04§	0.01	0.07	0.03§	0.01	0.04	-0.03	-0.06	0.00	0.44		-0.36	1.23
New COVID-19 deaths†	0.85§	0.45	1.26	-2.48	-11.82	6.86	0.41	-0.06	0.88	-4.44§	-8.86	-0.02	1.43§	0.89	1.97	80.24§		21.68	138.80
GDP per capita‡	0.01§	0.00	0.02	0.07§	0.05	0.10	0.00	-0.03	0.03	0.00	-0.03	0.02	-0.03§	-0.04	-0.02	0.02		-0.06	0.10
Health expenditure‡	-1.09§	-1.35	-0.83	-0.29	-0.72	0.14	-1.06§	-1.71	-0.42	-1.00§	-1.81	-0.19	-0.93§	-1.37	-0.50	5.08§		1.68	8.47
Public expenditure on health‡	-0.21§	-0.27	-0.16	-0.40§	-0.52	-0.29	-0.12§	-0.21	-0.02	-0.02	-0.16	0.13	-0.02	-0.14	0.09	-0.26§		-0.51	-0.02
September 2020 to February 2021*																			
New COVID-19 cases†	0.17§	0.11	0.23	0.03§	0.01	0.05	0.07§	0.04	0.11	0.01§	0.00	0.01	0.07	-0.12	0.26	0.81§		0.37	1.24
New COVID-19 deaths†	-4.21§	-6.24	-2.19	1.05§	0.17	1.94	6.15§	3.89	8.40	0.51§	0.18	0.83	6.29	-10.14	22.72	6.86		-11.69	25.40
GDP per capita‡	0.02§	0.00	0.05	0.06§	0.04	0.08	0.01	-0.01	0.03	0.04§	0.02	0.05	0.03	-0.06	0.12	0.04§		0.02	0.05
Health expenditure‡	-1.24§	-1.68	-0.79	-1.62§	-2.17	-1.07	-1.57§	-2.75	-0.40	-0.31	-0.77	0.15	-6.34§	-8.94	-3.75	-0.50		-1.01	0.00
Public expenditure on health‡	-0.08	-0.21	0.05	-0.05	-0.13	0.02	0.05	-0.13	0.22	-0.24§	-0.33	-0.16	-0.33§	-0.58	-0.08	-0.39§		-0.46	-0.32
March 2021 to August 2021*																			
New COVID-19 cases‡	<0.01§	<0.01	0.01	0.03§	0.02	0.04	-0.01	-0.02	0.00	0.02§	0.01	0.03	0.13§	0.06	0.19	0.05§		0.01	0.08
New COVID-19 deaths‡	-0.95	-1.93	0.03	0.23	-0.06	0.51	1.96§	0.65	3.27	-0.49§	-0.76	-0.22	-3.83§	-6.84	-0.81	0.80		-4.13	5.74
GDP per capita†	0.08§	0.06	0.10	0.07§	0.05	0.09	0.00	-0.02	0.02	0.00	-0.01	0.01	-0.03	-0.08	0.03	0.03§		0.02	0.05
Health expenditure†	0.80§	0.33	1.27	-1.31§	-2.00	-0.62	-0.74	-1.48	0.00	0.63§	0.25	1.01	-2.76	-5.63	0.11	-0.64		-1.31	0.03
Public expenditure on health†	0.04	-0.06	0.14	0.00	-0.12	0.11	0.15§	0.02	0.28	-0.08§	-0.14	-0.02	-0.23§	-0.35	-0.11	-0.73§		-0.82	-0.65

ME – marginal effects from three separate fractional regression models for each 6-mo period, GDP – gross domestic product, CI – confidence interval

*Epi-week 10 to 35 in 2020 used for March 2020 to August 2020 period; epi-week 36 in 2020 to epi-week 8 in 2021 used for September 2020 to February 2021 period; epi-week 9 in 2021 to epi-week 34 in 2021 used for March 2021 to August 2021 period.

†One new case per 100 000 population; one new death per 100 000 population.

‡1% increase in GDP per capita; 1% additional share of GDP on health expenditure; 1% additional share of public expenditure on health.

§*P* < 0.05.

GDP per capita did not have consistent associations with stringency across all periods within any region, and the pattern of associations was not the same for any two regions. Higher health expenditure was associated with less stringent policies within each region in the March 2020 to August 2020 and September 2020 to February 2021 periods, except for South-East Asia. Higher health expenditure was mostly associated with less stringent policies but was only associated with more stringency in Europe (March 2021 to August 2021). Public expenditure on health consistently showed a negative association with policy stringency in the Western Pacific region for all periods, but only for some periods in other regions.

## DISCUSSION

To our knowledge, this is the first study to show the epidemiological drivers of COVID-19 policy stringency globally and within regions, adjusting for GDP per capita, health expenditure, and public expenditure on health. We have empirically demonstrated that countries and territories responded more rapidly and strongly to the rise in new cases and deaths early in the COVID-19 pandemic than in subsequent periods. New deaths were more strongly associated with stringent policies than new cases across all periods examined and in most regions. Despite African countries having weaker mortality surveillance systems [30], the association between new deaths and policy stringency was more potent in Africa early in the pandemic than in all other regions except for the Western Pacific. Health expenditure as a fraction of GDP and public spending on health was negatively associated with policy stringency globally, but with regional variations. GDP per capita did not have consistent patterns of associations globally or within regions.

Prior assessments have focused on understanding the effects of public health policies on COVID-19 epidemiological and clinical outcomes. The current evidence shows that public health policies are essential for controlling SARS-COV-2 transmission and will likely remain so, despite the increased accessibility of safe and effective COVID-19 vaccines [8]. We found that the overall stringency of policies decreased globally, coinciding with an increasing vaccine coverage worldwide, especially in high- and upper-middle-income countries. For example, COVID-19 cases surged in Seychelles after the sudden relaxation of public health policies for NPIs in April 2021, before Omicron was detected, despite a rapid scale-up of vaccination coverage to 60% [31]. Maintaining stringent policies for prolonged periods also comes at a cost. School closures showed associations with adverse psychosocial outcomes among children during the pandemic [32]. More broadly, the COVID-19 pandemic has negatively affected mental health [33], food insecurity [34], and decreased the utilization of primary health care services [35].

An important gap in the current literature is the lack of an empirical understanding of the influence of reported new COVID-19 cases and deaths on policy stringency. Our study provides new evidence in the global landscape and within geographic regions and its empirical understanding of COVID-19 policy drivers can help public health officials anticipate policy responses in future health emergencies, especially before vaccines become available. Mathematical models have been relied upon to predict the course of health emergencies, but these models have not accounted for the dynamic drivers of governments’ policy responses. Our results may improve the parametrization of mathematical models in health emergencies.

A prior study showed that countries with weaker health systems, such as those in low- and middle-income countries, were quicker to implement more stringent policies early in the pandemic [3]. High-income countries with more significant investments in their health care systems were more equipped to absorb the initial shocks of the COVID-19 burden. Globally, however, health care systems have been overwhelmed at peak times of hospitalizations during each COVID-19 wave [36]. We found that places with less health expenditure as a share of their GDP were more inclined to enact stringent policies during the COVID-19 pandemic.

Countries with higher health expenditures may rely more on their capacity to provide medical care to patients instead of instituting stringent policies. Still, additional research is needed to test this hypothesis. We also found that greater public expenditure on health was associated with less strict policies. Additional research is necessary to understand how economic and political factors intertwine in potentially impacting policy stringency in countries with greater public spending on health. Unpredictable political and economic considerations make it difficult to anticipate how governments react to this pandemic's policymaking decisions or future health emergencies. Additional research is also needed to understand to what extent countries with weak mortality surveillance may have opted for less stringent policies because of their under-detection of COVID-19 deaths.

Our study has several limitations. First, we cannot discern the enforcement of or adherence to government policies. Such limitation does not necessarily weaken our results or their interpretations, given that our research focused on understanding how epidemiologic and socioeconomic factors influence policy reactions. Another limitation is that factors we did not account for in our models may have confounded, moderated, or mediated policy stringency. For example, we did not account for the potential influence of geographic proximity between countries. Moreover, hospitalizations and hospital bed capacities likely mediated policy stringency, but standardized global data on these variables are lacking. Finally, we cannot make any causal inferences regarding policy stringency.

Despite these limitations, our findings provide a new understanding of how COVID-19 cases and deaths may have impacted policy stringency. Our methods offer a framework for other researchers to build upon to get a more holistic picture of the drivers of policy stringency. Notably, the inclusion and exclusion of different combinations of socioeconomic covariates from our models did not have a meaningful impact on our findings regarding the associations of new cases and deaths with policy stringency. For instance, our models remained stable with or without public expenditure on health.

## CONCLUSIONS

The association of new COVID-19 deaths and cases with policy stringency waned as the pandemic progressed. However, new deaths were more strongly associated with stringent policies than new cases. Findings from this study reinforce the need for enhanced mortality surveillance to ensure policy alignment during health emergencies. Timely detection of excess deaths may help policymakers communicate their policy decisions more effectively to the public. We found that countries with lesser health expenditure as a share of their GDP were inclined to enact more stringent policies. However, countries with greater health expenditure may also be influenced by confounding or moderating factors that our study did not consider. An important implication of our findings is for public health authorities to tailor their support to policymakers to ensure alignment between the stringency of policies and the public health requirements of the health emergency. Our methods and findings may help public health officials anticipate, and shape policy responses in future health emergencies, especially before vaccines become accessible to the public.

## Additional material:


Online Supplementary Document
