# Supplementary material for: Drivers of COVID-19 policy stringency in 175 countries and territories: COVID-19 cases and deaths, gross domestic products per capita, and health expenditures
Source: J Glob Health. 2022 Dec 17;12:05049. doi: 10.7189/jogh.12.05049 (PMC9758449; doi:10.7189/jogh.12.05049)
Supplement: Online Supplementary Document [file jogh-12-05049-s001.pdf]

ONLINE SUPPLEMENTARY DOCUMENT

Title: Drivers of COVID-19 policy stringency in 175 countries and territories: COVID-19 cases and deaths, gross domestic products per capita, and health expenditures

Authors: Mohamed F. Jalloh, Zangin Zeebari2, Sophia A. Nur, Dimitri Prybylski, Aasli A. Nur, Avi J. Hakim, Maike Winters, Laura C. Steinhardt, Wangeci Gatei, Saad B. Omer, Noel T. Brewer, Helena Nordenstedt

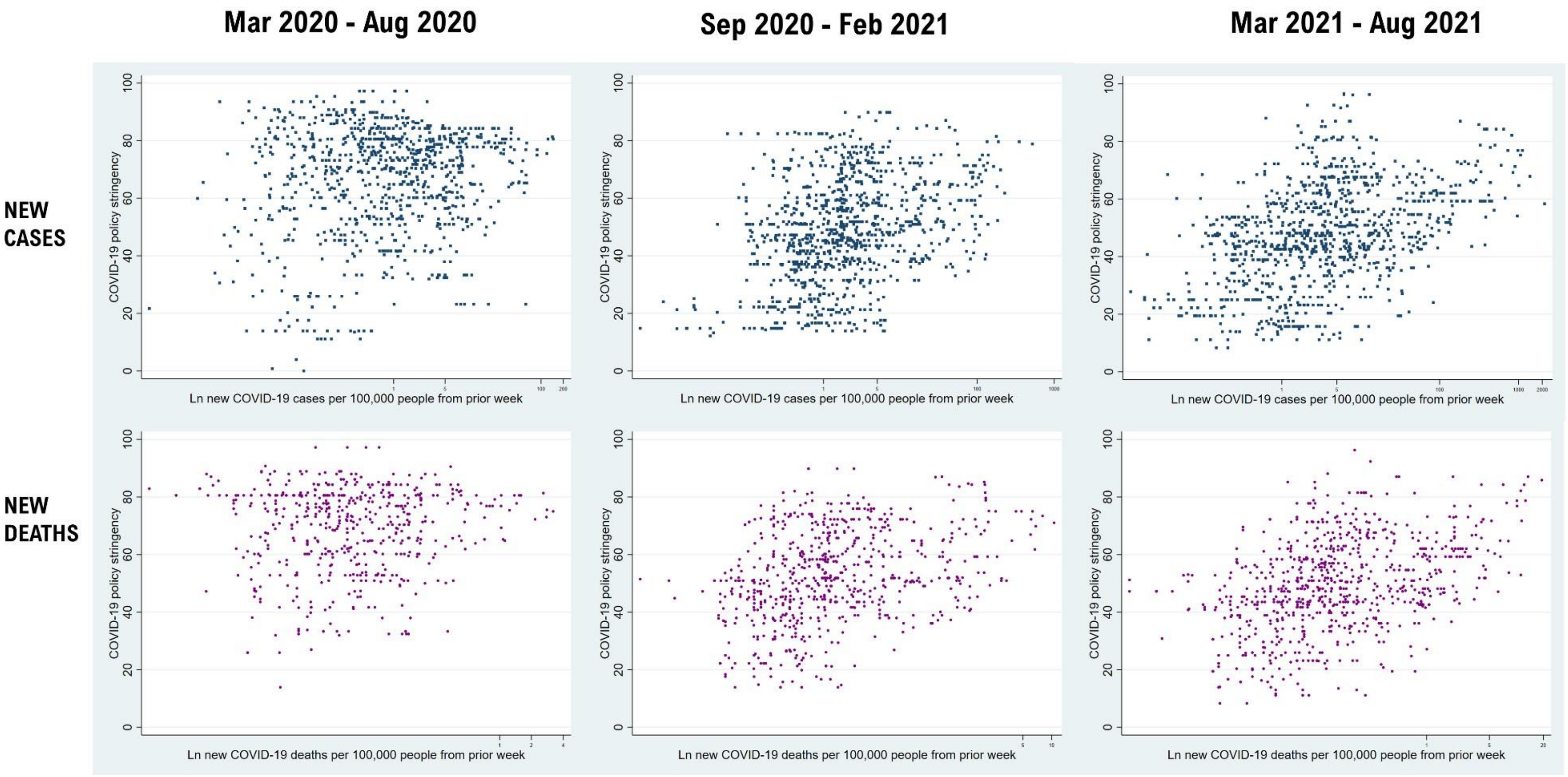

Figure S1. Association of stringency with logarithm of new cases and deaths per 100,000 in Africa, March 2020—August 2021.

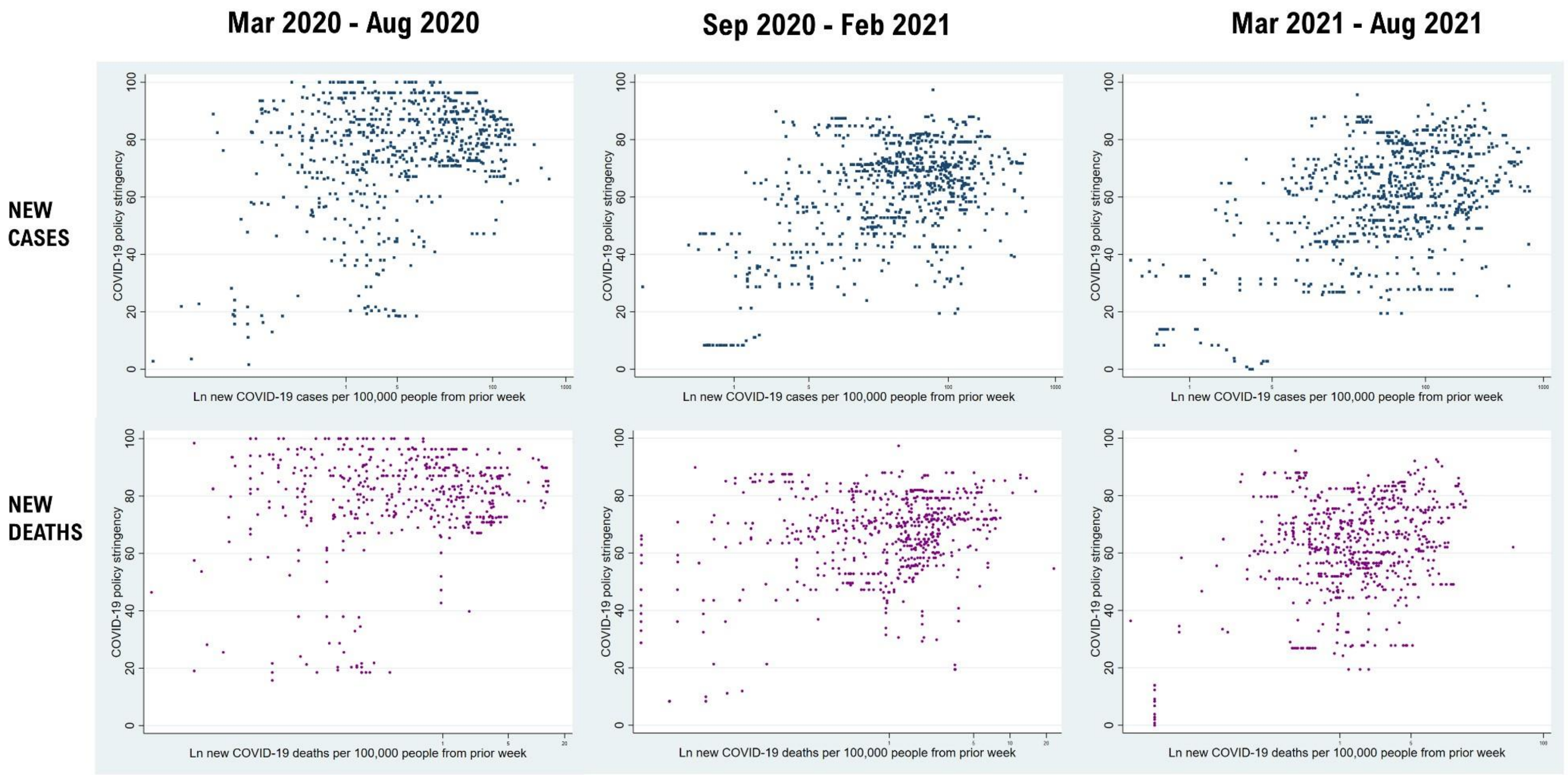

Figure S2. Association of stringency with the logarithm of new cases and deaths per 100,000 in the Americas region, March 2020—August 2021.

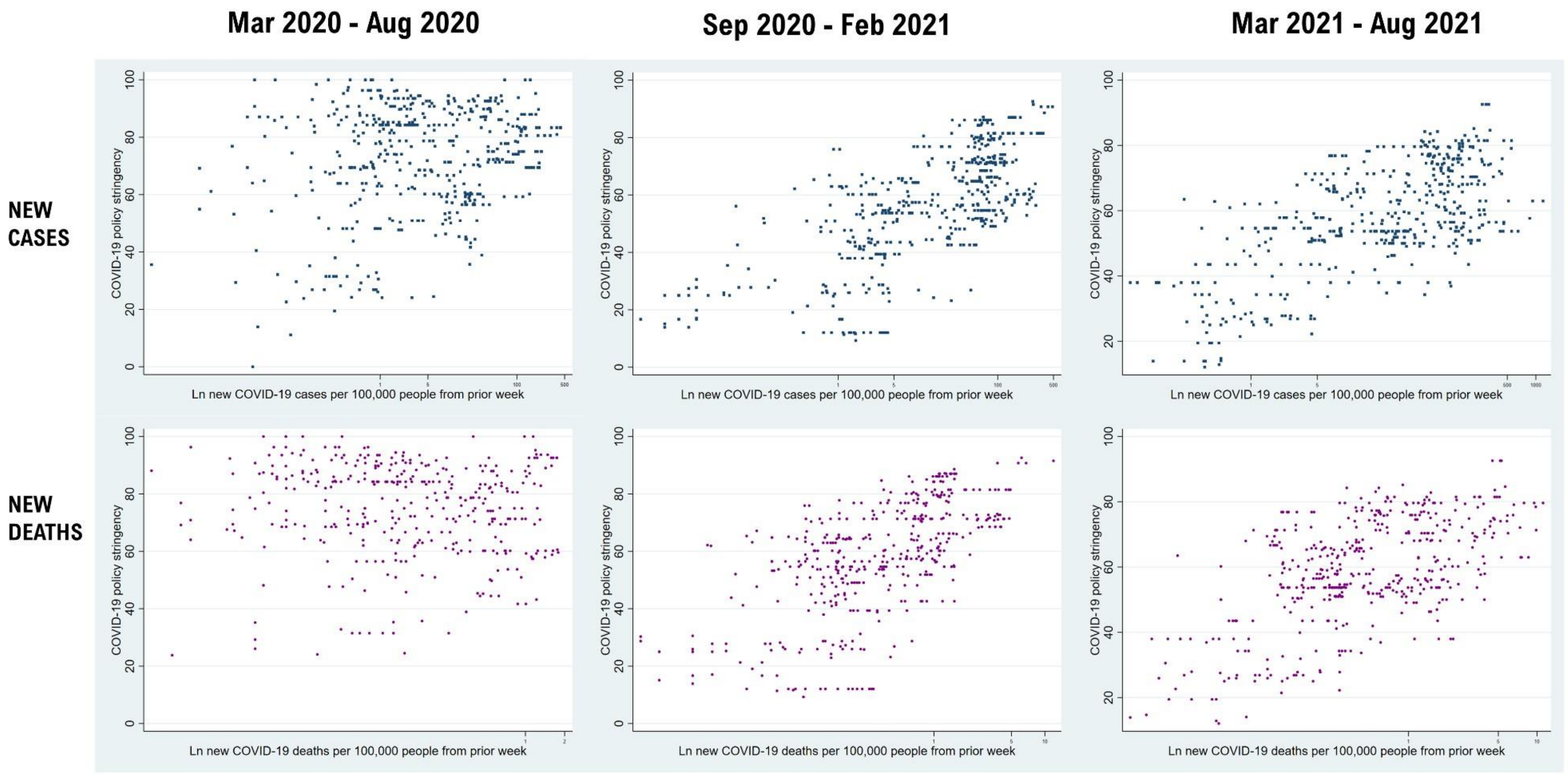

Figure S3. Association of stringency with the logarithm of new cases and deaths per 100,000 in Eastern Mediterranean region, March 2020—August 2021.

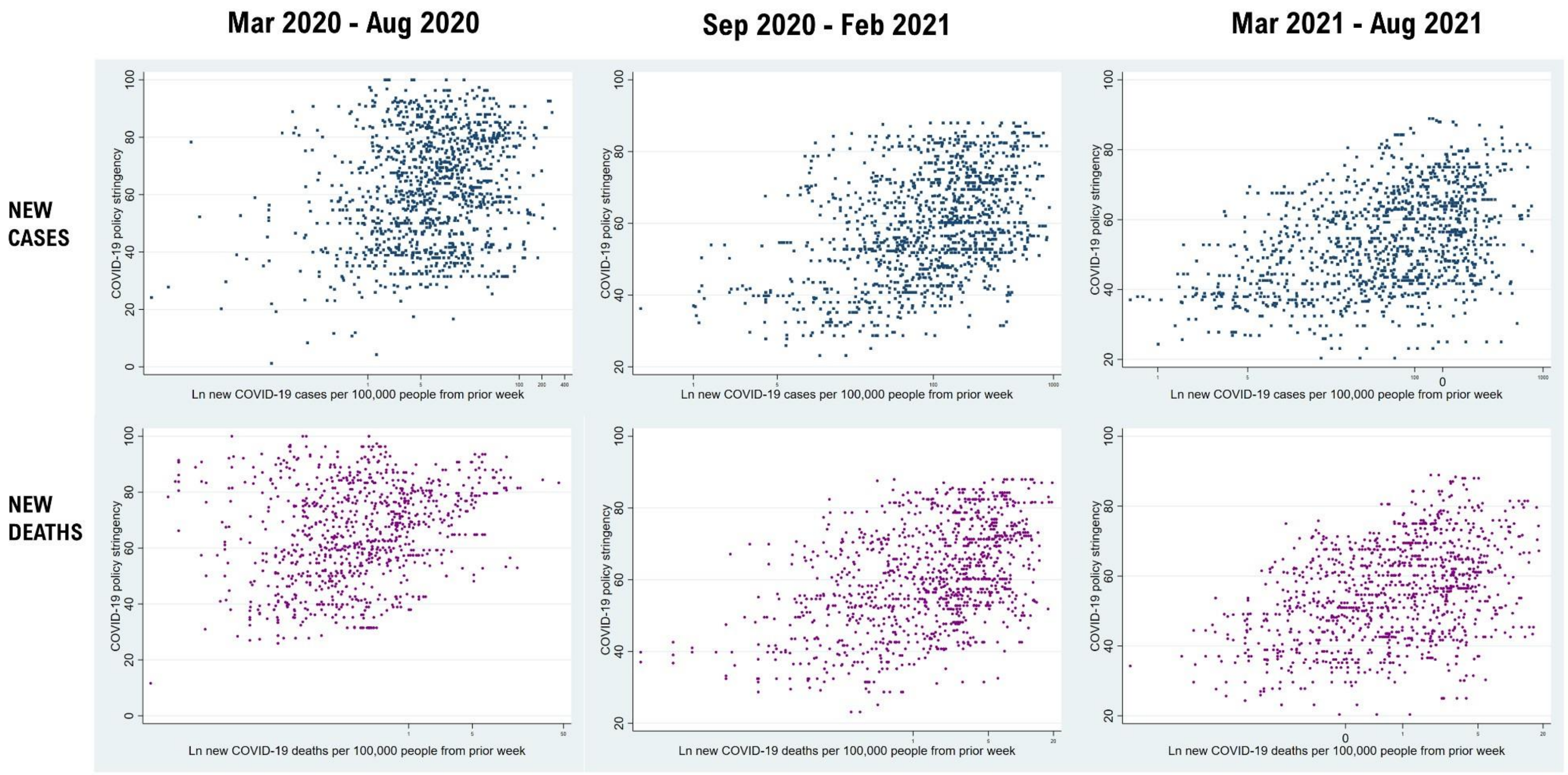

Figure S4. Association of stringency with logarithm of new cases and deaths per 100,000 in the European region, March 2020—August 2021.

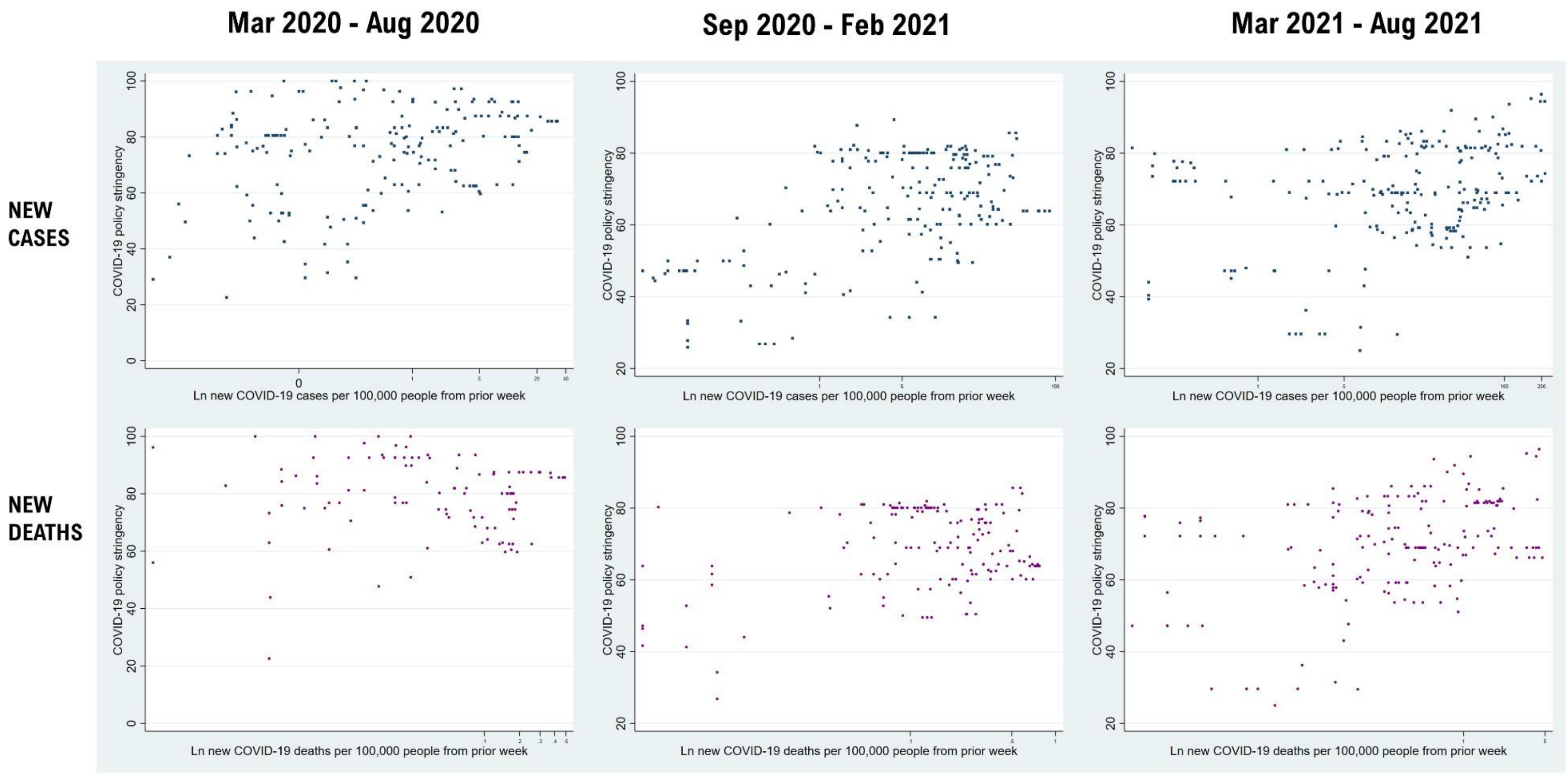

Figure S5. Association of stringency with logarithm of new cases and deaths per 100,000 in the South-east Asia region, March 2020—August 2021.

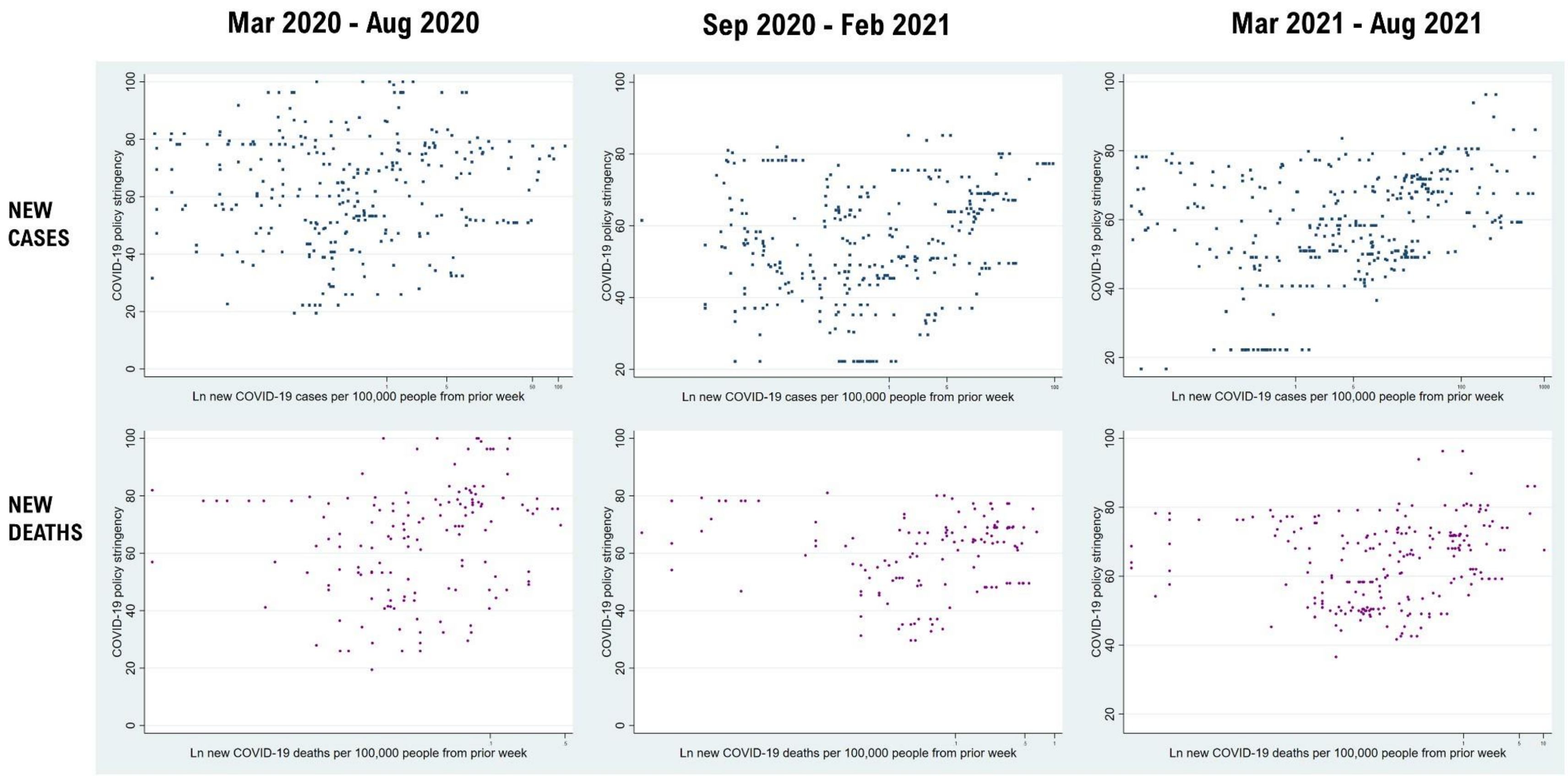

Figure S6. Association of stringency with logarithm of new cases and deaths per 100,000 in the Western Pacific region, March 2020—August 2021.
